# In Silico Network Toxicology, Molecular Docking, and Multi‐Level Bioinformatics Reveal Methyl Eugenol‐Induced Hepatocellular Carcinoma Mechanisms in Humans

**DOI:** 10.1002/cam4.70768

**Published:** 2025-05-15

**Authors:** Fuat Karakuş, Zübeyde Tanrıverdi, Burak Kuzu

**Affiliations:** ^1^ Department of Pharmaceutical Toxicology Faculty of Pharmacy, Van Yuzuncu Yil University Van Turkey; ^2^ Department of Pharmaceutical Toxicology Faculty of Pharmacy, Ağrı İbrahim Çeçen University Ağrı Turkey; ^3^ Department of Pharmaceutical Chemistry Faculty of Pharmacy, Van Yuzuncu Yil University Van Turkey

**Keywords:** *CCNB1*, cell cycle, hepatocellular carcinoma, in silico, methyl eugenol

## Abstract

**Background:**

Methyl eugenol (ME), a natural compound found in various essential oils, has recently been classified as a Group 2A carcinogen by the International Agency for Research on Cancer.

**Methods:**

This study aims to investigate the potential molecular mechanisms and underlying ME‐induced hepatocellular carcinoma (HCC) in humans using network toxicology, molecular docking, and integrative bioinformatics approaches, including transcriptomic and survival analyses of human HCC datasets.

**Results:**

Enrichment analysis highlighted significant associations with pathways related to steroid metabolic processes, extracellular exosomes, and diverse binding activities. KEGG pathway enrichment further implicated metabolic pathways in ME‐induced HCC development. Integration of STRING and Cytoscape analyses identified 14 hub targets, including key proteins such as *AURKB*, *CCNB1*, *CDK1*, and *PLK1*. Molecular docking studies demonstrated weak binding affinities of ME to these targets compared to their specific inhibitors. However, microarray data and survival analyses of human HCC samples revealed that *AURKB*, *CCNB1*, *CDK1*, and *PLK1* are upregulated in HCC, with higher expression levels correlating with poorer overall survival, particularly for *CCNB1*.

**Conclusions:**

These findings suggest that ME exposure may enhance the expression of these genes in hepatocytes, disrupting the cell cycle and promoting proliferation. This study provides valuable insights into the molecular mechanisms of ME‐induced HCC in humans and highlights potential therapeutic targets, such as *CCNB1*, for further investigation.

## 1 Introduction

Methyl eugenol (ME) is a naturally occurring compound found in the essential oils of several herbs and spices, including basil, lemongrass, and fennel. This liquid is distinguished by its clove‐like aroma and can be synthesized through the methylation of eugenol [1, 2, 3]. Historically, ME has been utilized as a flavoring agent in a range of food products and consumer items, such as cosmetics, personal care products, insect attractants, and even as an anesthetic in veterinary settings [3, 4, 5].

The primary sources of human exposure to ME primarily stem from consuming food and beverages. Additionally, personal care products containing ME, along with insect repellents, contribute to the overall exposure of the general population. It is anticipated that exposure levels for the general populace are below 1 μg/kg body weight per day from each source [3]. In June 2023, ME was classified as “probably carcinogenic to humans” (Group 2A), based on “sufficient” evidence of carcinogenicity from experimental animals and “strong” mechanistic evidence, including studies conducted on humanized mice and supported by mechanistic analyses of exposed humans [3]. The majority of well‐designed in vivo chronic toxicity and carcinogenicity studies subject to this IARC (2023) report indicate that the highest incidence of cancer observed was hepatocellular carcinoma (HCC) [3, 4, 6, 7, 8].

In 2022, liver cancer was responsible for over 750,000 deaths worldwide, making it the third leading cause of cancer‐related fatalities, following lung and colorectal cancers. It was also the sixth most commonly diagnosed cancer, with an estimated 865,000 new cases and 757,948 deaths reported, according to GLOBOCAN statistics. HCC represents approximately 75%–85% of all primary liver cancers [9]. Thus, gaining a comprehensive understanding of the mechanisms underlying HCC is essential. In this context, examining the impact of ME on human HCC is crucial for assessing health risks and developing appropriate safety protocols.

In recent years, computational toxicology has become an essential tool for investigating the intricate interactions between chemical substances and biological systems. This field utilizes computational models to integrate various omics data, offering a comprehensive understanding of how toxins disrupt cellular networks and contribute to disease development. Furthermore, bioinformatics techniques, such as pathway analysis, play a pivotal role in identifying critical targets and mechanisms associated with toxicity, thus providing insights into the molecular foundations of adverse effects. These methodologies present a holistic perspective on chemical toxicity, aiding in the identification of biomarkers and potential therapeutic strategies [10, 11, 12].

This study investigates the potential mechanisms linking HCC in humans to ME by employing network toxicology, molecular docking, and bioinformatics. Additionally, by integrating gene expression data from HCC patients, the research aims to validate key targets identified as significant contributors to human HCC. This approach not only deepens our understanding of the carcinogenic mechanisms associated with ME in humans but also aids in the development of strategies for risk assessment and management.

## 2 Methods

### 2.1 Investigated Compound

In this study, Methyl eugenol (ME) (CAS number: 93‐15‐2) was utilized to explore the mechanisms underlying HCC.

### 2.2 Curation of ME Targets

The Simplified Molecular Input Line Entry System (SMILES) notation and molecular structure of ME were sourced from the PubChem database (https://pubchem.ncbi.nlm.nih.gov) [13]. Subsequently, potential targets of ME specific to *Homo sapiens* were identified using the Comparative Toxicogenomics Database (CTD; https://ctdbase.org) [14], the SwissTargetPrediction tool (http://www.swisstargetprediction.ch) [15], ChEMBL (https://www.ebi.ac.uk/chembl/) [16], and STITCH (http://stitch.embl.de) [17]. All these tools were accessed on November 10–15, 2024. The structural data from all sources were meticulously cross‐validated for accuracy. All identified targets were then consolidated and deduplicated, with their gene nomenclature standardized through the UniProt database (https://www.uniprot.org), culminating in the creation of a comprehensive ME target library.

### 2.3 Construction of HCC‐Associated Targets

Comprehensive searches were conducted across several databases, including Disease Gene Network (DisGeNET; https://www.disgenet.com) [18], Online Mendelian Inheritance in Man (OMIM; https://www.omim.org) [19], GeneCards (https://www.genecards.org) [20], and the CTD [14] to identify relevant targets associated with HCC. All these tools were accessed on November 15–20, 2024. The keywords “hepatocellular carcinoma” and “hepatoma” were utilized in these searches, leading to the establishment of a refined library of HCC targets, free from duplications. Additionally, a Venn diagram was created using the Jvenn tool (https://jvenn.toulouse.inrae.fr/app/index.html) [21] to illustrate the intersecting targets between the ME and HCC datasets. This subset of overlapping targets was considered potential candidates involved in ME‐induced HCC.

### 2.4 Gene Ontology and KEGG Pathway Enrichment Analysis of Potential Targets

The biological mechanisms associated with potential targets in ME‐induced HCC were investigated through Gene Ontology (GO) and Kyoto Encyclopedia of Genes and Genomes (KEGG) pathway enrichment analyses. This analysis was performed using the Database for Annotation, Visualization, and Integrated Discovery (DAVID; https://davidbioinformatics.nih.gov/tools.jsp) (accessed on December 1, 2024) [22], which offers comprehensive information on gene functions, encompassing biological processes (BPs), cellular components (CCs), and molecular functions (MFs) [22]. Furthermore, KEGG pathway enrichment analysis was conducted to identify significant pathways related to the potential targets of ME‐induced HCC. Results with a false discovery rate (FDR) of < 0.05 were deemed statistically significant, and the top 10 terms from both GO and KEGG analyses were visualized using SRplot (https://www.bioinformatics.com.cn/en) [23].

### 2.5 Construction of Protein–Protein Interaction (PPI) Networks and Identification of Hub and Key Targets

The potential targets associated with ME‐induced HCC were analyzed using the Search Tool for the Retrieval of Interacting Genes/Proteins (STRING; https://string‐db.org) (accessed on December 1, 2024) [24] to map their corresponding proteins and interaction networks. The analysis parameters were rigorously defined, focusing exclusively on *Homo sapiens* , requiring a minimum interaction score of > 0.9 (indicating the highest confidence), and setting an FDR stringency value to ensure a concentrated examination of active proteins linked to target genes [24].

Data from STRING were subsequently imported into Cytoscape software (version 3.10.1) [25] for the calculation of topological properties of network nodes and edges, visualization of molecular connections, and construction of the PPI network diagram. Four distinct algorithms—maximum clique centrality (MCC), maximum neighborhood component (MNC), Degree centrality, and the Molecular Complex Detection (MCODE) method through the cytoHubba plugin—were employed to evaluate node significance scores and identify critical hub targets. Targets that were recognized by at least two of these algorithms were classified as hub targets. By integrating essential topological parameters, such as degree centrality, betweenness centrality, and closeness centrality, the top hub targets with the highest cumulative values were determined to be the key targets.

### 2.6 Pathway Enrichment Analysis of Hub Targets

Pathway enrichment analysis was also performed on the hub targets utilizing both the Metascape (https://metascape.org/gp/index.html#/main/step1) [26] and DAVID [22]. This analysis aimed to thoroughly explore the signaling pathways and biological processes implicated in HCC pathologies mediated by the hub targets, thereby highlighting critical mechanisms. The enrichment results were ultimately visualized as Sankey and dot plots via SRplot [23] to enhance the effective interpretation and presentation of the findings.

### 2.7 Molecular Docking

Molecular docking studies were conducted using AutoDock 4.2 software [27] to evaluate the binding affinity and interaction patterns between ME and its targets. The molecular structure of ME was retrieved from PubChem [13], and the protein structures were obtained from the RCSB Protein Data Bank (www.rcsb.org). Crystal co‐ligands and ME were modeled using GaussView 5.0 and optimized via the DFT method in Gaussian 03, employing the B3LYP method with the 6–31G basis set.

The docking simulations utilized the following PDB IDs: AURKB: 4C2V [28], CCNB1‐CDK1 complex: 6GU3 [29], and PLK1: 3THB [30]. Prior to docking, solvents, water molecules, and unwanted ligands were removed, while polar hydrogen atoms were added along with Gasteiger partial charges and Kollman charges. A grid box was positioned to encompass the structural regions of each protein, allowing unrestricted molecular motion.

The coordinate centers for docking were determined as follows: *x* = 6.113, *y* = −24.108, *z* = 4.608 for 4C2V; *x* = 95.405, *y* = −71.699, *z* = 184.016 for 6GU3; and *x* = −44.196, *y* = 11.229, *z* = 9.219 for 3THB. The docking pockets were defined as a cubic space of 50 × 50 × 50 Å for both 4C2V and 6GU3, and 40 × 40 × 64 Å for 3THB, with grid points spaced 0.375 Å apart. Additionally, the rotatable bonds of the compounds were adjusted.

The Lamarckian genetic algorithm was employed for docking, with 10 runs and a population size of 300 for all simulations. To validate the docking process, the native ligand was re‐docked and compared to its experimentally solved crystal geometry. The conformation with the highest receptor affinity was selected, and the binding energy was determined.

### 2.8 Microarray Data and Survival Analysis

To validate the findings of this research, a differentially expressed genes (DEGs) analysis was conducted between human HCC samples and paired normal liver tissue using the Gene Expression Profiling Interactive Analysis (GEPIA2; http://gepia2.cancer‐pku.cn/#index) web server [31]. DEGs were defined based on the criteria of |Log_2_FC| > 1 and a *q*‐value cutoff < 0.05, using the Linear Models for Microarray Data (LIMMA) method with an adjusted *p*‐value (Benjamini and Hochberg FDR) in GEPIA2. These DEGs were then compared with the hub genes, and the overlapping key genes were visualized alongside the DEGs in a volcano plot.

Survival analysis was performed using the GEPIA2 web server [31] to evaluate the clinical significance of key genes based on their expression levels. Kaplan–Meier survival curves were generated to examine the association between key gene expression and survival rates in patients with HCC. Log‐rank *p*‐values and hazard ratios (HRs) were calculated to quantify the differences.

## 3 Results

### 3.1 Acquisition of Targets in ME‐Induced HCC

**FIGURE 1:**
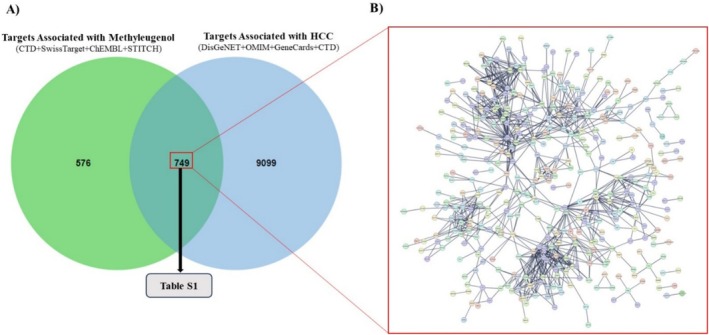
Venn diagram and PPI network of potential targets for ME‐induced HCC. (A) The green circle represents 1325 ME targets, while the blue circle corresponds to 9848 HCC‐related targets. The overlapping region identifies 749 potential targets (Table S1) associated with both ME and HCC, suggesting their relevance in ME‐induced HCC. (B) The PPI network, generated from STRING with a high‐confidence score of 0.9, illustrates interactions among these potential targets.

In this study, 1325 ME targets related to ME were curated by integrating data from the CTD, SwissTargetPrediction, ChEMBL, and STITCH. Concurrently, 9848 targets strongly associated with HCC were identified from the DisGeNET, GeneCards, OMIM, and CTD databases. By employing an integrative Venn diagram approach, 749 intersecting targets (Table S1) were obtained and regarded as potential targets involved in ME‐induced HCC (Figure 1A).

### 3.2 PPI Network of Potential Targets

The potential targets linked to ME‐induced HCC were mapped onto a PPI network using the STRING database. This resulted in a highly interconnected network comprising 746 nodes and 1000 edges, with an average node degree of 2.68 and a PPI enrichment *p*‐value of less than 1.0 × 10^−16^. The PPI network derived from STRING is depicted in Figure 1B.

### 3.3 GO And KEGG Pathway Mapping of Potential Targets

**FIGURE 2:**
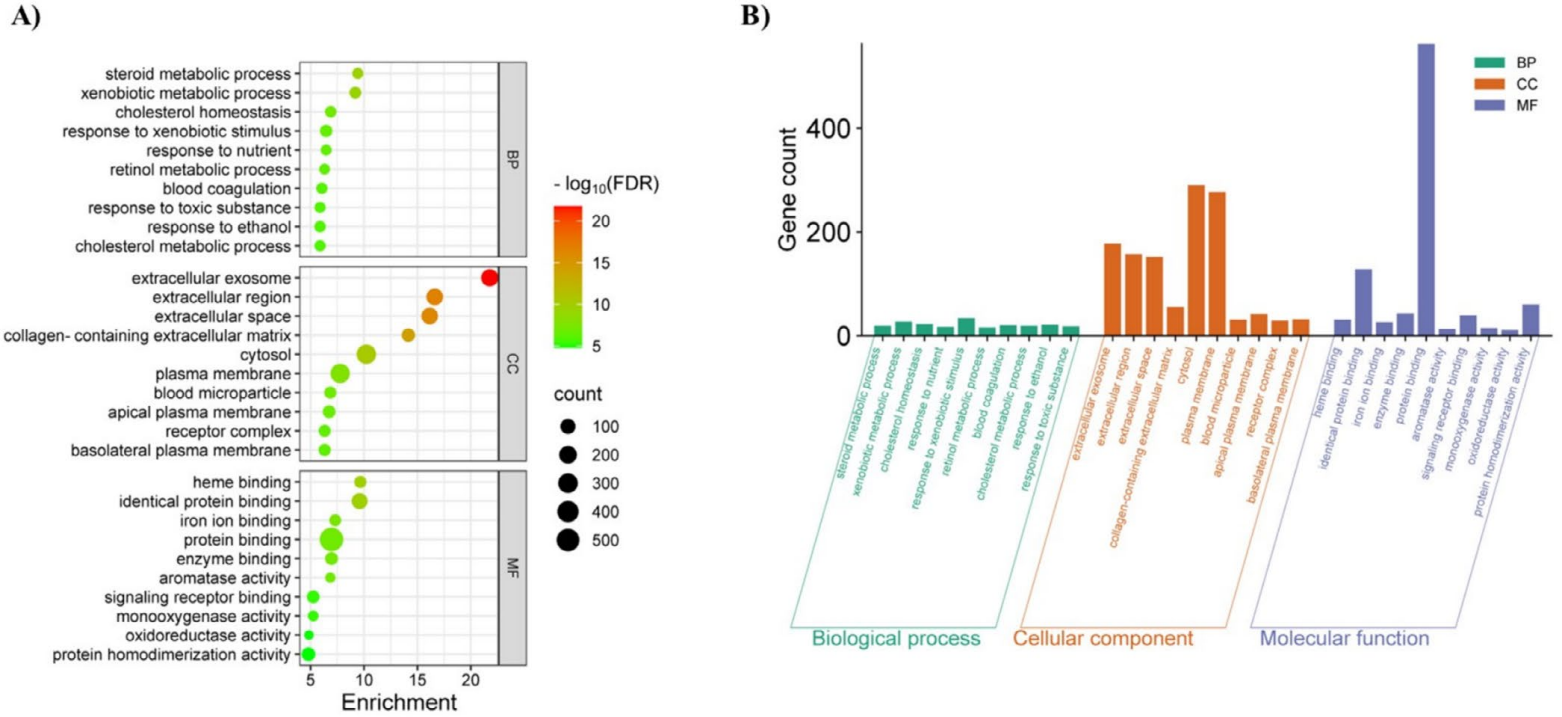
GO enrichment analysis for 749 potential targets. (A) The size of each bubble represents gene expressions within a specific term, while the color saturation corresponds to FDR values, with smaller values indicating greater enrichment significance. (B) The histogram illustrates the top 10 enriched terms in each GO category. The height of each bar reflects the gene count, representing the enrichment level in the corresponding category.

The analysis encompassed 749 potential targets, with GO analysis conducted using the DAVID database. The results identified significant GO terms, comprising 584 biological processes (BPs), 103 cellular components (CCs), and 166 molecular functions (MFs). The prioritization of GO terms was guided by FDR values, and the top ten most enriched terms with the lowest FDR scores for each category were visually represented in enrichment analysis diagrams (Figure 2A,B). The potential targets exhibited noteworthy enrichment in pathways related to the “steroid metabolic process” under BPs. CC analysis indicated functional roles in extracellular exosomes, while the enriched MFs were significantly linked to “heme binding”. The GO enrichment results are illustrated through bubble and histogram diagrams in Figure 2A,B, with comprehensive pathway terms listed in Table S2.

**FIGURE 3:**
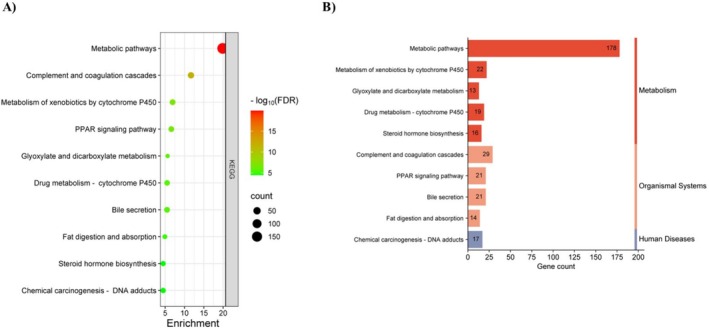
KEGG pathway enrichment analysis of 749 potential targets. (A) The bubble plot displays the top 10 most enriched KEGG signaling pathways, ranked by their FDR values. Each bubble represents a pathway, with its size proportional to the number of enriched genes. The color intensity indicates the significance of enrichment. (B) The histogram illustrates the frequency and significance of enrichment for each pathway. The length of each bar corresponds to the gene count, representing the enrichment score.

In addition, KEGG pathway analysis was conducted on the 749 potential targets using the DAVID database, revealing 119 enriched pathways associated with these targets. A bubble chart and categorical histogram highlighting the most enriched KEGG signaling pathways were generated (Figure 3). Importantly, a strong association between “metabolic pathways” and ME‐induced HCC was observed. Detailed pathway terms are provided in Table S3.

### 3.4 Hub and Key Targets Identification

**FIGURE 4:**
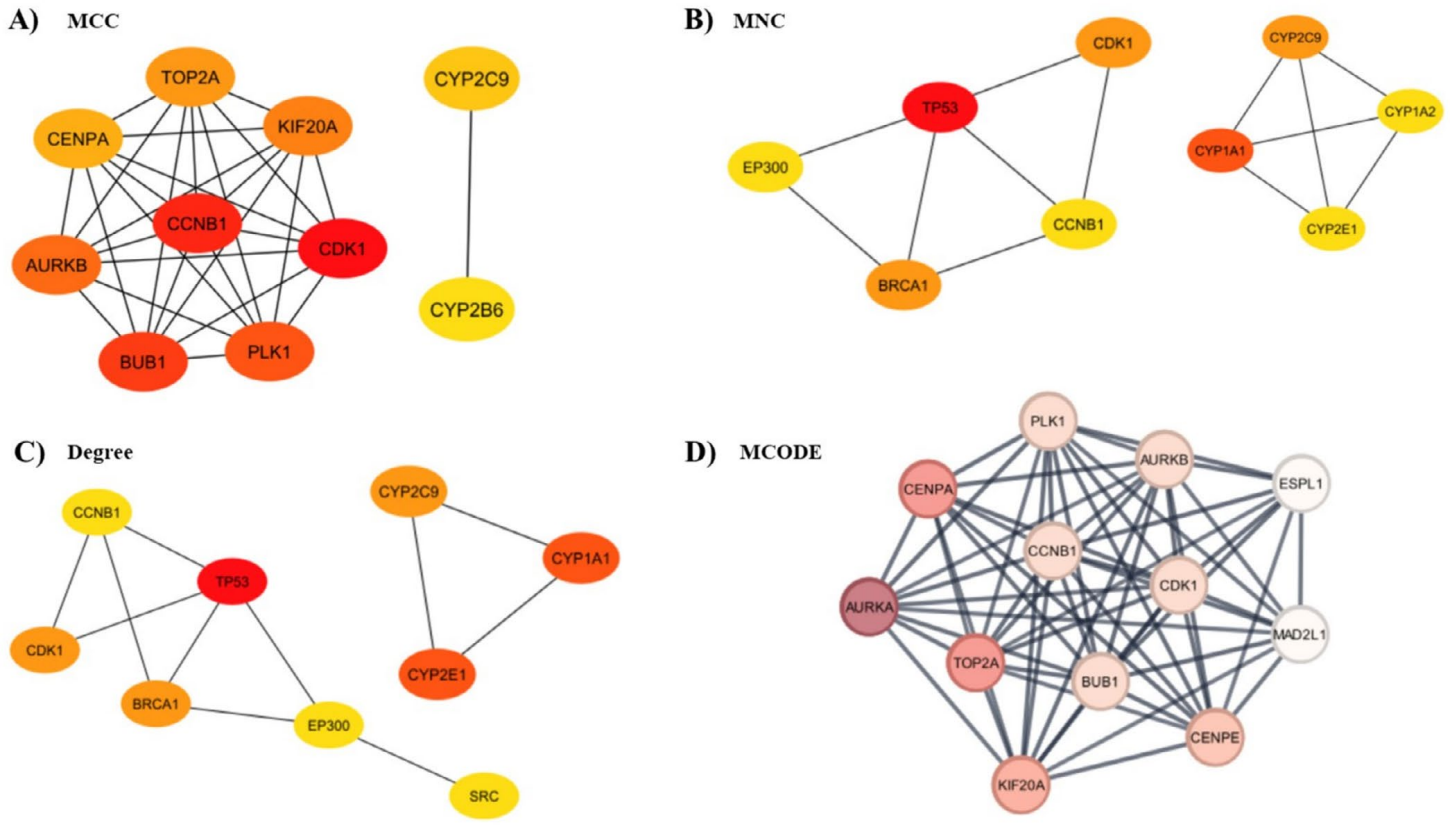
PPI network of hub and key targets. (A–D) Hub target candidates were identified using MCC, MNC, Degree, and MCODE algorithms. The color intensity of the nodes correlates with their calculated degree, with more intense coloring indicating higher degree values.

Following the construction of the PPI network encompassing 749 potential targets (Figure 1B), a variety of complementary algorithms were applied to the STRING PPI network. These included MCC, MNC, Degree, and MCODE, all sourced from the cytoHubba plugin of Cytoscape (Figure 4), in order to identify highly connected hub targets.

**FIGURE 5:**
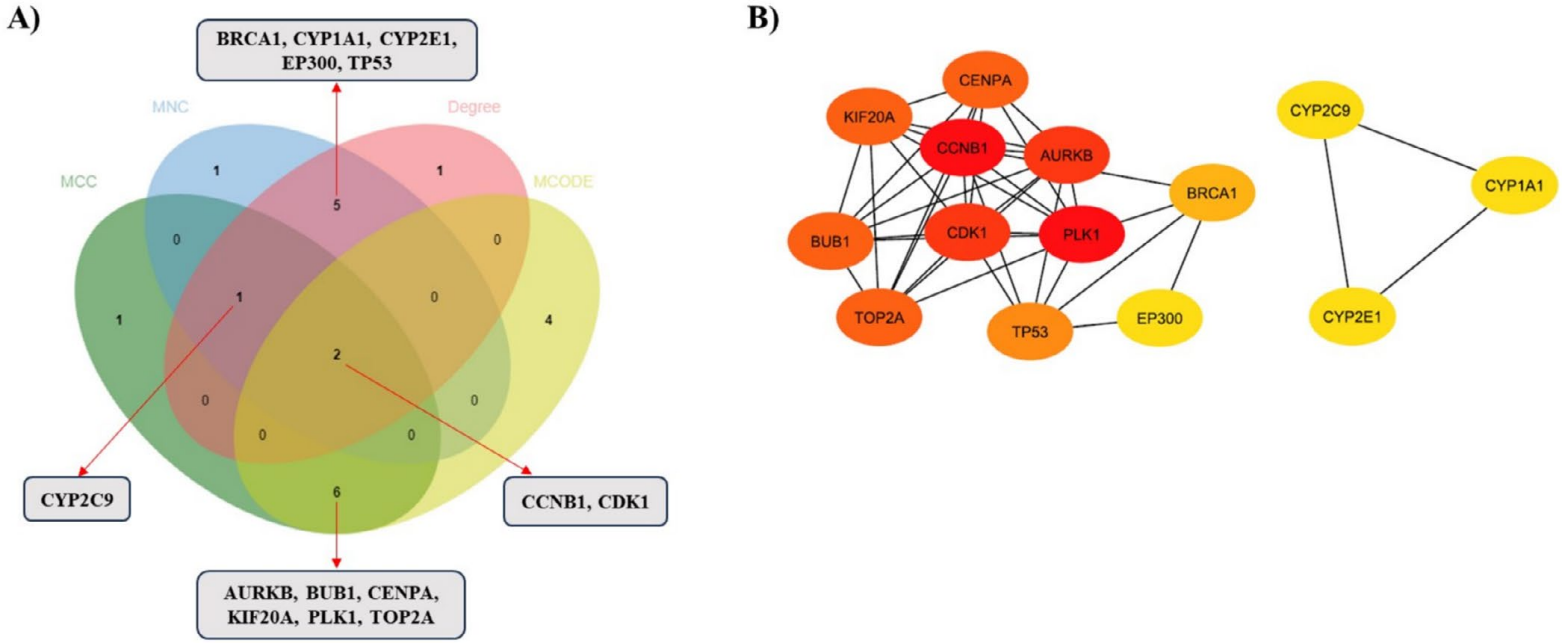
(A) Hub targets identified by at least two of the MCC, MNC, Degree, and MCODE algorithms. (B) PPI network of hub targets with key targets identified through centrality analysis. The color intensity of the nodes correlates with their calculated degree, with more intense coloring indicating higher degree values.

Targets identified as hubs by at least two of these methods were selected, resulting in a subset of 14 targets closely associated with ME‐induced HCC (Figure 5). These 14 targets were also ranked according to their degree scores (Table 1).

**TABLE 1:** Centrality analysis of 14 hub targets.

Gene	Degree	Closeness centrality	Betweenness centrality
*CCNB1*	9	0.9091	0.1111
*PLK1*	9	0.9091	0.1111
*AURKB*	8	0.8333	0.0370
*CDK1*	8	0.8333	0.0370
*BUB1*	7	0.7143	0.0000
*CENPA*	7	0.7143	0.0000
*KIF20A*	7	0.7143	0.0000
*TOP2A*	7	0.7143	0.0000
*TP53*	6	0.7143	0.1407
*BRCA1*	4	0.6250	0.0519
*CYP1A1*	2	1.0000	0.0000
*CYP2C9*	2	1.0000	0.0000
*CYP2E1*	2	1.0000	0.0000
*EP300*	2	0.4545	0.0000

A focused PPI network diagram was then developed to illustrate the interactions among these hub targets. Notably, the top four hub targets, ranked by degree, were identified as key targets, including Aurora kinase B (*AURKB*), G2/mitotic‐specific cyclin‐B1 (*CCNB1*), Cyclin‐dependent kinase 1 (*CDK1*), and Polo‐like kinase 1 (*PLK‐1*) (Figure 5 and Table 1).

**FIGURE 6:**
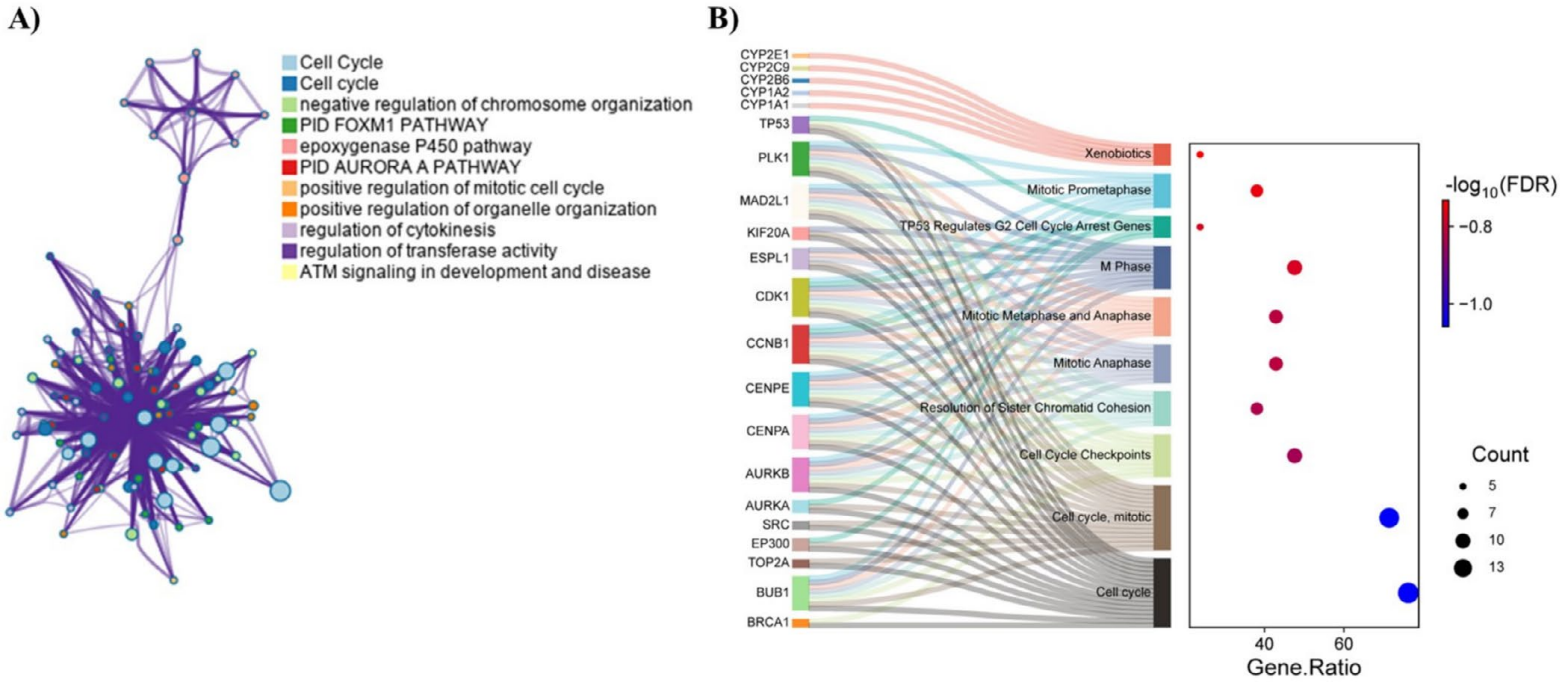
Pathway enrichment analysis of 14 hub targets. (A) Cluster graphs from Metascape associated with the 14 hub targets. (B) The Sankey diagram and dot plot display the top 10 most enriched REACTOME signaling pathways, ranked by their FDR values from DAVID. Each dot represents a specific pathway and is linked with gray lines. The size of each dot corresponds to the number of enriched genes.

The 14 identified hub targets were subsequently subjected to a comprehensive pathway enrichment analysis using the Metascape and DAVID databases. This analysis revealed a total of 101 significant signaling pathways, with a particular emphasis on cell‐cycle pathways. A cluster graph was generated from Metascape (Figure 6A), and a Sankey‐dot blot of the top 10 enriched REACTOME terms from DAVID (Figure 6B), ranked by FDR values, was visualized using SR_plot_. Detailed pathway terms can be found in Table S4. The analysis indicated that the predominant pathways associated with the 14 hub targets in ME‐induced HCC were closely linked to cell‐cycle regulation.

### 3.5 Molecular Docking of ME to Key Targets

To assess the affinity of ME for the four key targets—*CCNB1*, *CDK1*, *PLK1*, and *AURKB*—molecular docking analysis was performed. The protein structures of human *AURKB*, the *CCNB1*–*CDK1*–*CKS2* complex, and PLK1 bound to their respective inhibitors were retrieved from the Protein Data Bank. Using AutoDock 4.2 tools, the binding poses and interactions of ME with these targets were analyzed, and binding energy values were obtained, as detailed in Table 2. The results indicated that ME exhibited weak to moderate affinity for all four targets compared to their respective inhibitor (control) compounds. These findings suggest that ME may not function as an inhibitor for these targets.

**TABLE 2:** Binding energies of ME and inhibitors against target proteins.

Target	Compounds	Binding energy (kcal/mol)	Predicted inhibitory concentrations (μM)
*AURKB* (PDB ID: 4C2V)	Barasertib (Control)	−7.82	1.84
	ME	−4.92	249.45
*CCNB1*–*CDK1*–*CKS2* complex (PDB ID: 6GU3)	AZD5438 (Control)	−10.04	0.04
	ME	−5.18	159.68
*PLK1* (PDB ID: 3THB)	MLN0905 (Control)	−11.18	0.006
	ME	−5.38	114.67

### 3.6 Results of DEGs and Survival Analyses

**FIGURE 7:**
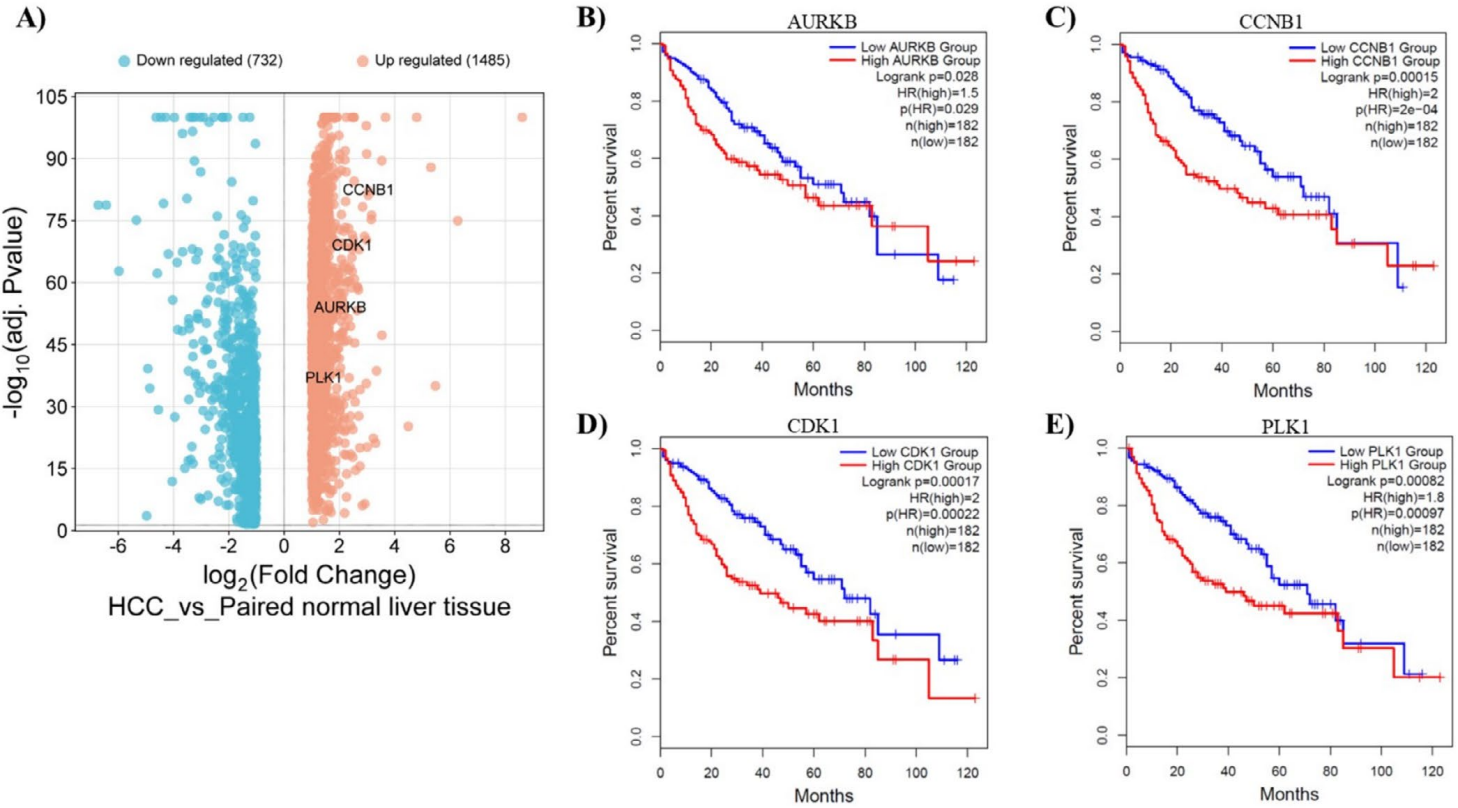
(A) DEGs between human HCC and paired normal liver tissues. (B–E) Overall survival rates of HCC patients associated with the key genes. Kaplan–Meier curves for *AURKB*, *CCNB1*, *CDK1*, and *PLK1*, respectively.

Using GEPIA2, a comparison of 369 human HCC samples with 50 paired normal liver tissues identified a total of 2217 DEGs, including 1485 upregulated genes and 732 downregulated genes. This analysis revealed that several key genes—specifically *AURKB*, *CCNB1*, *CDK1*, and *PLK1*—were significantly upregulated in HCC patients compared to normal tissues (Figure 7A).

Kaplan–Meier survival curves were constructed for these key genes, and survival rates between high‐ and low‐expression groups were compared using the log‐rank test. Notably, high expression of *AURKB* was associated with poorer survival outcomes (log‐rank *p*: 0.028; HR: 1.5, *p*: 0.029) (Figure 7B). For *CCNB1*, elevated expression significantly worsened overall survival (log‐rank *p*: 0.00015; HR: 2, *p*: 2 × 10^−4^) (Figure 7C). Similarly, high expression levels of *CDK1* were correlated with decreased survival (log‐rank *p*: 0.00017; HR: 2, *p*: 0.00022) (Figure 7D). Additionally, high expression of *PLK1* also indicated significantly reduced survival rates (log‐rank *p*: 0.00082; HR: 1.8, *p*: 0.00097) (Figure 7E).

## 4 Discussion

This study provides evidence linking the Group 2A carcinogen ME to potential mechanisms of HCC in humans. By integrating network toxicology and bioinformatics approaches, 749 potential targets were identified (Figure 1). Enriched GO terms highlighted the significance of steroid metabolic processes, extracellular exosomes, and heme binding in ME‐induced HCC (Figure 2). KEGG pathway enrichment analysis further suggested associations with various metabolic pathways (Figure 3).

The liver is pivotal in the metabolism of steroids, including glucocorticoids, mineralocorticoids, and sex hormones. Disruptions in steroid metabolic pathways, commonly observed in HCC, often arise from altered enzymatic activity (such as cytochrome enzymes like CYP3A4) and dysregulation of steroid hormone receptors (including estrogen and androgen receptors) [32]. Such disruptions facilitate tumor progression. For instance, the androgen receptor is overexpressed in approximately 74% of HCC cases in men and 38% in women, correlating with advanced disease and poor prognosis [33]. Similarly, nuclear estrogen receptor α and β expressions are significantly elevated in HCC tissues compared to adjacent non‐cancerous tissues [34].

Centrality analysis of PPIs using MCC, MNC, Degree, and MCODE algorithms identified *AURKB*, *CCNB1*, *CDK1*, and *PLK1* as critical proteins in ME‐induced HCC (Figures 4 and 5, Table 1). These proteins play crucial roles in the cell cycle (Figure 6A,B), act as key regulators of mitosis, particularly during mitotic entry, progression, and spindle checkpoint functions [35]. However, molecular docking studies revealed that ME has weak binding affinities for these proteins (Table 2), suggesting it is unlikely to act as their direct inhibitor. GEO analysis confirmed that these genes are significantly upregulated in HCC patients (Figure 7A), and survival analysis revealed their higher expression levels correlate with poorer overall survival, particularly for *CCNB1* (Figure 7B–E).

*AURKB*, a serine/threonine kinase and key member of the aurora kinase family, orchestrates crucial mitotic events through the chromosomal passenger complex. Beyond its established role in chromosome segregation and cytokinesis [36], recent evidence reveals *AURKB*'s oncogenic potential in HCC through multiple mechanisms. Its upregulation induces hyperpolyploidy in hepatocytes, triggering their transformation into HCC cells [37]. Furthermore, *AURKB* phosphorylates multiple downstream targets, including histone H3 at Ser10, which leads to chromatin condensation and altered gene expression patterns characteristic of HCC progression. Our survival analysis supports *AURKB*'s role as a prognostic biomarker for ME‐induced HCC (Figure 7B).

*CCNB1* emerges as particularly significant in our analysis, demonstrating the strongest correlation with poor survival outcomes among the identified hub genes (Figure 7C). As the regulatory subunit of *CDK1*, *CCNB1* plays a pivotal role in orchestrating mitotic entry through the formation of the *CCNB1*–*CDK1* complex. This complex's activity is tightly regulated under normal conditions, but its dysregulation in HCC leads to several critical oncogenic consequences. First, aberrant *CCNB1* expression disrupts the precise timing of mitotic entry, leading to premature cell division and chromosomal instability [38]. Second, *CCNB1* overexpression prevents the proper activation of DNA damage checkpoints, allowing cells with genomic abnormalities to continue dividing. Recent studies have revealed that *CCNB1* also interacts with p53 pathways, potentially suppressing apoptotic responses in damaged cells [39, 40]. The robust prognostic value of *CCNB1* in HCC, confirmed by multiple independent studies [41, 42, 43], along with its central position in our network analysis, establishes it as a prime therapeutic target for ME‐induced HCC.

*CDK1*, activated through its association with *CCNB1*, serves as the master regulator of the G2/M transition and mitotic progression. Our analysis reveals its consistent upregulation in HCC patients, suggesting its crucial role in ME‐induced carcinogenesis. *CDK1* dysregulation promotes tumor development through multiple mechanisms: it drives unscheduled mitosis, leading to genomic instability and aneuploidy [44]; phosphorylates key survival proteins, preventing apoptosis of damaged cells [45]; and contributes to therapy resistance through interaction with DNA damage repair pathways. Recent studies demonstrate that disrupting the *CCNB1*–*CDK1* complex effectively retards tumorigenesis [46, 47], positioning *CDK1* as a promising therapeutic target in HCC [48, 49].

*PLK1*, a serine/threonine kinase, coordinates multiple critical aspects of mitosis, including centrosome maturation, spindle assembly, and cytokinesis [50]. Our findings of *PLK1* overexpression in HCC align with previous studies showing its essential role in tumor cell proliferation [51, 52]. *PLK1*'s oncogenic effects extend beyond its mitotic functions; it phosphorylates multiple cancer‐relevant substrates, promoting cell survival and metastasis. Recent evidence indicates that *PLK1* downregulation not only inhibits HCC cell survival but also sensitizes cells to conventional chemotherapy [53], suggesting its potential as a therapeutic target.

The coordinated upregulation of these four hub genes reveals a complex network of cell cycle dysregulation in ME‐induced HCC. *CCNB1* stands out as particularly significant due to its central role in the *CCNB1*–*CDK1* complex and its robust prognostic value. The interaction between these proteins creates a feed‐forward loop promoting uncontrolled cell division: *CCNB1*–*CDK1* complex activation leads to *PLK1* phosphorylation, which in turn promotes AURKB activity, further driving cell cycle progression. This complex interplay suggests that targeted therapeutic strategies might benefit from simultaneously addressing multiple components of this network, with *CCNB1* serving as a primary target due to its central regulatory role and strong correlation with patient outcomes.

## 5 Conclusion

This study employed integrative computational analyses to uncover the molecular mechanisms underlying ME‐induced HCC, identifying 749 potential targets through network toxicology and bioinformatics approaches. Our systematic analysis revealed 14 hub proteins, with particular emphasis on *AURKB*, *CCNB1*, *CDK1*, and *PLK1*, which emerged as critical regulators of cell cycle progression and mitotic processes. Through GEO analysis and survival studies, we validated that these genes are significantly overexpressed in HCC patients and correlate with poor clinical outcomes. Notably, *CCNB1* emerged as a particularly significant mediator, demonstrating the strongest association with patient survival and serving as a central node in the regulatory network through its interaction with *CDK1* and influence on downstream pathways.

The coordinated upregulation of these hub genes, particularly the *CCNB1*–*CDK1* complex, suggests that ME exposure drives HCC development through systematic dysregulation of the hepatocyte cell cycle, leading to enhanced cell proliferation and chromosomal instability. While molecular docking studies indicated weak direct binding between ME and these proteins, their consistent dysregulation points to complex downstream effects of ME exposure on cell cycle regulation. These findings not only advance our understanding of ME‐induced hepatocarcinogenesis but also identify *CCNB1* as a promising prognostic biomarker and potential therapeutic target. Future studies should focus on validating these computational predictions and exploring the therapeutic potential of targeting *CCNB1* and its associated pathways in ME‐induced HCC.

## Author Contributions

**Fuat Karakuş:** conceptualization, methodology, data curation, formal analysis, investigation, visualization, writing – original draft preparation, writing – reviewing and editing. **Zübeyde Tanrıverdi:** investigation, data curation, formal analysis, visualization, writing – original draft preparation. **Burak Kuzu:** investigation, software, visualization, writing – original draft preparation.

## Conflicts of Interest

The authors declare no conflicts of interest.

## Supporting information


Data S1.

## Data Availability

The authors confirm that the data supporting the findings of this study are available within the article [and/or] its Supporting Information.
