# Supplementary material for: In Silico Network Toxicology, Molecular Docking, and Multi‐Level Bioinformatics Reveal Methyl Eugenol‐Induced Hepatocellular Carcinoma Mechanisms in Humans
Source: Cancer Med. 2025 May 15;14(10):e70768. doi: 10.1002/cam4.70768 (PMC12079025; doi:10.1002/cam4.70768)
Supplement: Supplementary file 1 — Data S1. [file CAM4-14-e70768-s001.docx]

**Supplementary Material**

***In Silico* Network Toxicology,** **Molecular Docking, and Multi-Level Bioinformatics Reveal Methyl Eugenol-Induced Hepatocellular Carcinoma Mechanisms in Humans**

**Supplementary Table Captions**

**Table S1.** Potential targets involved in ME-induced HCC, obtained from CTD, SwissTarget, ChEMBL, and STITCH databases.

**Table S2.** The enrichment chart of the GO function analysis for 749 potential targets ([DAVID](https://david.ncifcrf.gov/tools.jsp)).

**Table S3.** KEGG pathway enrichment analysis of 749 potential targets ([DAVID](https://david.ncifcrf.gov/tools.jsp)).

**Table S4.** REACTOME pathway enrichment analysis of 14 hub targets ([DAVID](https://david.ncifcrf.gov/tools.jsp)).

**Table S1.** Potential targets involved in ME-induced HCC, obtained from CTD, SwissTarget, ChEMBL, and STITCH databases.

| AASS, ABAT, ABCA1, ABCB11, ABCB4, ABCC11, ABCC6, ABCG5, ABCG8, ACAA1, ACACB, ACAT2, ACHE, ACMSD, ACOT1, ACOT12, ACOX2, ACSL1, ACSL4, ACSL5, ACSM3, ACSM5, ACSS1, ACSS2, ACYP2, ADAMTS17, ADAMTSL3, ADCY5, ADGRG7, ADH1B, ADH4, ADH6, ADK, ADRA1A, AFM, AGPAT2, AGR2, AGT, AGXT, AHSG, AIG1, AKAP12, AKR1B1, AKR1B10, AKR1C4, AKR1D1, AKR7A3, ALB, ALDH1A1, ALDH3A1, ALDH4A1, ALDH6A1, ALDOB, ALOX15, ALOX5, AMBP, ANG, ANGPTL3, ANGPTL8, ANK1, ANK2, ANKRD1, ANXA10, ANXA3, APBA1, APBB1IP, APCS, APMAP, APOA1, APOA4, APOA5, APOC3, APOH, APOM, AQP4, AQP9, AR, ARB2A, ARG1, ARRB1, ARSB, ART3, ASGR1, ATM, ATP8B4, ATR, AURKA, AURKB, AZGP1, B3GNT3, B4GALNT1, BAAT, BABAM2, BAIAP2L2, BANK1, BATF3, BAX, BBOX1, BCKDHB, BCL2L15, BDH1, BDKRB2, BHMT, BMF, BMPR1B, BRCA1, BRCA2, BRCC3, BRD9, BRINP1, BRPF1, BUB1, C2CD4A, C4BPA, C4BPB, C5, C6, C9, CA12, CA2, CA3, CA9, CACNA1D, CADM2, CADPS2, CALB2, CAMK1D, CAMK4, CASP3, CAT, CBR3, CBX5, CCBE1, CCDC146, CCDC171, CCL16, CCN2, CCNB1, CCNG1, CD22, CD44, CD9, CDC7, CDCA3, CDH1, CDK1, CDK14, CDKAL1, CDKN1A, CDKN2A, CDO1, CEACAM1, CEMIP, CENPA, CENPE, CFAP52, CFB, CFHR1, CFHR3, CFHR4, CHEK1, CHEK2, CHRM1, CISH, CLDN14, CMSS1, CNTN1, COBL, COL18A1, COL5A2, COMMD1, COMMD10, CPA4, CPN1, CPQ, CPS1, CPVL, CREBBP, CRELD2, CRP, CRYBG2, CTH, CTNNA3, CTNNB1, CTSC, CTSK, CTSS, CTSV, CX3CR1, CXCL13, CXXC4, CYP11B1, CYP11B2, CYP19A1, CYP1A1, CYP1A2, CYP1B1, CYP2A6, CYP2B6, CYP2C19, CYP2C8, CYP2C9, CYP2D6, CYP2E1, CYP3A7, CYP4A11, CYP4B1, CYP7A1, CYP7B1, CYP8B1, DAB1, DCAF11, DCXR, DDC, DDR2, DENND1A, DGAT1, DGAT2, DHRS2, DHRS9, DHTKD1, DIRAS3, DISC1, DLG2, DNAH5, DOCK1, DOCK8, DPYD, DPYS, DSC2, DUSP9, DYNC2H1, EDIL3, EEF1A2, EFCAB11, EGFR, EGR1, EHHADH, ELMO1, ELP4, EP300, EPHX2, EPM2A, EPS8L3, ERBB4, EREG, ERP27, ESPL1, ESPN, ESRRG, EXOC3L4, EXOC4, EYA2, EYA4, F11, F12, F2RL2, F7, F9, FABP1, FAM167A, FAM72B, FAM83D, FARS2, FASN, FBLIM1, FBP1, FBXO43, FER, FETUB, FGA, FGB, FGFR2, FGGY, FGL2, FHIT, FILIP1, FILIP1L, FOSL1, FOXA3, FRAS1, FST, FTO, FUT6, FXYD5, FYN, G6PC1, GABRA3, GABRB3, GABRG2, GAD1, GADD45B, GATM, GBA3, GC, GCDH, GCGR, GCNT3, GDF11, GDF15, GEM, GFRA1, GHR, GIPC2, GJA3, GLA, GLDC, GLIPR1, GLIPR2, GLS2, GLUD1, GLUL, GLYAT, GLYATL1, GLYCTK, GMDS, GNMT, GPAM, GPC2, GPC5, GPC6, GPD1, GPHN, GPM6A, GPR87, GPRC5A, GPT, GRAP, GREB1, GRID2, GRIP1, GRM5, GSTA1, GSTA2, GSTA3, GSTM1, GSTM2, GSTP1, GTDC1, GULP1, GYS2, H2AX, H2BC3, H2BC8, H3C1, HAGH, HAO2, HAVCR2, HDAC4, HHAT, HJV, HMCN1, HMGA1, HMGCR, HMGCS1, HMGCS2, HMMR, HMOX1, HNMT, HOMER3, HOXB9, HP, HPD, HPN, HPR, HPX, HRG, HSD17B2, ID2, IDI1, IER3, IGF1, IL16, IL1B, IL1RL1, IMPDH1, IMPG2, INCENP, INHBA, INHBC, INPP4B, INSIG1, IQGAP2, ITGA2, ITIH1, ITPR1, ITPR3, IYD, JAK1, JAK2, JUNB, KAT2A, KAT2B, KCND3, KDM8, KDR, KHK, KIF20A, KLB, KLC4, KLF12, KLF15, KLF6, KLKB1, KMO, KNG1, KRT20, KRT5, KRT6A, LAIR1, LASP1, LBP, LDB2, LDLRAD4, LFNG, LGALS4, LHPP, LIF, LIPC, LMO2, LRBA, LRP1B, LRP2, LRP8, LTBP1, LYPD1, MACROD2, MAD1L1, MAD2L1, MAGI1, MAN1C1, MARCHF1, MARCKS, MARCKSL1, MAT1A, MATN2, MB, MBL2, MDK, MDM2, MELTF, MFSD2A, MGLL, MGMT, MKI67, MLKL, MMP10, MMP12, MMUT, MOGAT2, MPDZ, MPG, MPP3, MSRA, MST1, MST1R, MSTN, MT1F, MTMR11, MTMR4, MTSS1, MTTP, MUC13, MUCL1, MVD, MVK, NBAS, NCAN, NEFL, NES, NEU4, NEUROG3, NFE2L2, NFE2L3, NGFR, NHEJ1, NKD2, NLRP6, NOS2, NOS3, NOSTRIN, NPY1R, NPY5R, NQO1, NQO2, NR0B2, NR1H4, NR1I3, NR3C1, NSDHL, NTS, NUBPL, NUDT1, OGDHL, OGG1, OLFML2A, ORM1, ORM2, OSBP2, OTC, OTUD7A, P2RX7, PAH, PAK5, PANK1, PARD3B, PARP1, PBLD, PCCA, PCDHAC2, PCK1, PCK2, PCSK9, PDE11A, PDE4D, PDE7B, PDE8B, PDLIM2, PDLIM7, PDZK1, PDZK1IP1, PECR, PEG10, PEPD, PERM1, PFKFB1, PFKP, PGLYRP2, PID1, PIK3AP1, PIK3CA, PIK3CB, PIPOX, PKD1L1, PKHD1, PKLR, PLA2G12B, PLA2G1B, PLCB1, PLCG2, PLG, PLK1, PLXNA3, PMS1, PMS2, PNPLA3, POLD3, PON1, PON3, PPARA, PPARD, PPARG, PPM1J, PPP1R1A, PPP1R3B, PPP2R2B, PRKAG2, PRKAR1B, PRKCE, PRKG1, PRKN, PRLR, PROC, PROCR, PROX1, PROZ, PRR11, PTGS1, PTH1R, PTHLH, PTPRD, RAB3IL1, RAC2, RAD50, RAD51B, RAD52, RAP1GAP, RASGRF2, RASGRP1, RASSF6, RB1, RBL2, RBP4, RBP5, REN, RETREG1, RFTN1, RGN, RGS5, RHBDF2, RHBG, RHPN1, RIDA, RNF125, RNF152, RNF157, RORC, RRAD, RSRC1, RYR2, S100A11, S100A14, S100A2, S1PR2, SAA2, SARDH, SCAPER, SCFD2, SCP2, SCRN1, SDK1, SELENBP1, SEPTIN4, SERINC2, SERPINA10, SERPINA11, SERPINA4, SERPINB2, SERPINC1, SERPIND1, SERPINE2, SERPINF1, SERPINF2, SFTPD, SH3PXD2A, SH3TC2, SHMT1, SHMT2, SIGLEC1, SLC10A1, SLC22A1, SLC22A10, SLC22A25, SLC22A7, SLC23A1, SLC25A33, SLC26A3, SLC27A5, SLC29A1, SLC2A2, SLC2A9, SLC37A4, SLC38A3, SLC38A4, SLC3A2, SLC47A1, SLC51A, SLC5A1, SLC5A11, SLC5A2, SLC6A4, SLC6A6, SLC7A11, SLC8A1, SLCO2B1, SLIT2, SMAD3, SMOC1, SMPD3, SMYD3, SNTB1, SORD, SORL1, SOX4, SOX5, SPARCL1, SPNS2, SPP1, SPP2, SPTB, SRC, SRD5A2, SREBF1, SRPX2, ST6GAL1, STC1, STX8, SULT1E1, SULT2A1, SYNPO2, SYT13, SYT7, SYT9, SYTL1, TAT, TBXAS1, TDO2, TEAD2, TF, TFPI2, TG, TGM2, THRB, THRSP, THSD7A, TINAGL1, TLN2, TLR6, TM6SF2, TMEM100, TMEM139, TMEM176A, TMPRSS6, TMSB10, TNFRSF10A, TNFRSF11A, TNFRSF11B, TNFRSF9, TNFSF4, TNS1, TOP2A, TOX3, TP53, TPK1, TRIM31, TRIM63, TSPAN15, TST, TTC28, TTN, TTPA, TUBA1A, TYK2, TYRO3, UBD, UGT2B15, UROC1, USP10, USP13, VDR, VEPH1, VPS13B, VSIG1, VSNL1, VTN, WNK3, WNT10B, WNT4, WNT5B, WNT6, WNT7B, WWOX, XDH, XRCC4, ZG16, ZG16B, ZNF521, ZNF726, ZRANB3 |
| --- |

**,**

**Table S2.** The enrichment chart of the GO function analysis for 749 potential targets ([DAVID](https://david.ncifcrf.gov/tools.jsp)).

| **Category** | **GO term** | **FDR** | **Gene count** |
| --- | --- | --- | --- |
| BP | steroid metabolic process | 3.80E-10 | 19 |
|  | xenobiotic metabolic process | 6.40E-10 | 27 |
|  | cholesterol homeostasis | 1.30E-07 | 23 |
|  | response to nutrient | 3.50E-07 | 17 |
|  | response to xenobiotic stimulus | 3.50E-07 | 34 |
|  | retinol metabolic process | 4.90E-07 | 16 |
|  | blood coagulation | 8.70E-07 | 20 |
|  | cholesterol metabolic process | 1.30E-06 | 19 |
|  | response to ethanol | 1.30E-06 | 21 |
|  | response to toxic substance | 1.30E-06 | 18 |
| CC | extracellular exosome | 1.70E-22 | 178 |
|  | extracellular region | 2.40E-17 | 157 |
|  | extracellular space | 7.00E-17 | 152 |
|  | collagen-containing extracellular matrix | 7.00E-15 | 55 |
|  | cytosol | 6.00E-11 | 290 |
|  | plasma membrane | 1.70E-08 | 277 |
|  | blood microparticle | 1.40E-07 | 31 |
|  | apical plasma membrane | 1.80E-07 | 42 |
|  | receptor complex | 4.80E-07 | 29 |
|  | basolateral plasma membrane | 4.80E-07 | 32 |
| MF | heme binding | 2.20E-10 | 31 |
|  | identical protein binding | 2.60E-10 | 128 |
|  | iron ion binding | 4.90E-08 | 26 |
|  | enzyme binding | 1.10E-07 | 43 |
|  | protein binding | 1.10E-07 | 563 |
|  | aromatase activity | 1.40E-07 | 13 |
|  | signaling receptor binding | 5.60E-06 | 39 |
|  | monooxygenase activity | 5.60E-06 | 15 |
|  | oxidoreductase activity | 1.40E-05 | 11 |
|  | protein homodimerization activity | 1.60E-05 | 60 |

**Table S3.** KEGG pathway enrichment analysis of 749 potential targets ([DAVID](https://david.ncifcrf.gov/tools.jsp)).

| **KEGG term** | **FDR** | **Gene count** |
| --- | --- | --- |
| Metabolic pathways | 1.30E-20 | 178 |
| Complement and coagulation cascades | 2.10E-12 | 29 |
| Metabolism of xenobiotics by cytochrome P450 | 1.20E-07 | 22 |
| PPAR signaling pathway | 2.60E-07 | 21 |
| Glyoxylate and dicarboxylate metabolism | 2.40E-06 | 13 |
| Drug metabolism - cytochrome P450 | 3.20E-06 | 19 |
| Bile secretion | 3.40E-06 | 21 |
| Fat digestion and absorption | 1.20E-05 | 14 |
| Steroid hormone biosynthesis | 3.40E-05 | 16 |
| Chemical carcinogenesis - DNA adducts | 3.40E-05 | 17 |

**Table S4.** REACTOME pathway enrichment analysis of 14 hub targets ([DAVID](https://david.ncifcrf.gov/tools.jsp)).

| **REACTOME term** | **FDR** | **Gene count** |
| --- | --- | --- |
| Cell Cycle | 3.60E-12 | 16 |
| Cell Cycle, Mitotic | 3.80E-12 | 15 |
| Cell Cycle Checkpoints | 9.90E-08 | 10 |
| Resolution of Sister Chromatid Cohesion | 1.60E-07 | 8 |
| Mitotic Anaphase | 2.70E-07 | 9 |
| Mitotic Metaphase and Anaphase | 2.70E-07 | 9 |
| M Phase | 9.70E-07 | 10 |
| TP53 Regulates Transcription of Genes Involved in G2 Cell Cycle Arrest | 1.20E-06 | 5 |
| Mitotic Prometaphase | 2.00E-06 | 8 |
| Xenobiotics | 4.00E-06 | 5 |
